# A multisite study of the overlap between symptoms and cognition in schizophrenia: Une étude multicentrique sur le chevauchement entre les symptômes et les troubles cognitifs chez les personnes atteintes de schizophrénie

**DOI:** 10.1177/07067437251387565

**Published:** 2025-10-22

**Authors:** Rafal M. Skiba, Abhijit M. Chinchani, Mahesh Menon, Martin Lepage, Katie M. Lavigne, Ashok Malla, Ridha Joober, Joel O. Goldberg, R. Walter Heinrichs, David J. Castle, Amy Burns, Michael W. Best, Susan L. Rossell, Sebastian Walther, Todd S. Woodward

**Affiliations:** 1 8166 BC Mental Health and Addictions Research Institute, Vancouver, British Columbia, Canada; 2 Department of Psychiatry, University of British Columbia, Vancouver, British Columbia, Canada; 3 Department of Psychiatry, University of British Columbia, Vancouver, British Columbia, Canada; 4 Douglas Research Centre, 5620 McGill University, Montreal, Québec, Canada; 5 Department of Psychiatry, 5620 McGill University, Montreal, Québec, Canada; 6 Department of Psychology, York University, Toronto, Ontario, Canada; 7 Department of Psychiatry, 7978 University of Tasmania, Hobart, Australia; 8 Department of Psychological Clinical Science, 33530 University of Toronto, Toronto, Ontario, Canada; 9 Centre for Mental Health, 3783 Swinburne University of Technology, Melbourne, Australia; 10 Department of Mental Health, St Vincent's Hospital, Melbourne, Australia; 11 Translational Research Center, University Hospital of Psychiatry and Psychotherapy, 27210 University of Bern, Bern, Switzerland; 12 Department of Psychiatry, Psychosomatics, and Psychotherapy, Center of Mental Health, University Hospital Würzburg, Germany

**Keywords:** schizophrenia, cognition, psychotic disorders, memory, verbal, memory, working, motor activity, communication disorders

## Abstract

**Objective:**

Cognitive impairment is a core feature of schizophrenia spectrum disorders. Our previous study on a first-episode psychosis cohort showed that symptoms related to impoverished/disorganized communication and motor impoverishment predicted verbal and working memory scores, respectively. This study aimed to explore those predictors in people across the range of illness chronicity.

**Methods:**

We employed iterative Constrained Principal Component Analysis (iCPCA) to investigate the relationship between 15 cognitive measures from the MATRICS battery, including processing speed, attention, working, verbal and nonverbal memory, reasoning, and problem-solving, and 27 Positive and Negative Syndrome Scale (PANSS) items in 198 outpatients from two sites in Australia and one in Canada. The iCPCA method was used to determine symptoms that reliably predict specific combinations of cognitive measures while controlling Type I errors.

**Results:**

We found that a verbal memory and learning component was predicted by the PANSS item *Lack of Spontaneity and Flow of Conversation*, and a visual attention/working memory component was linked to the PANSS item *Motor Retardation*.

**Conclusions:**

These accord with our previous findings in an early psychosis sample, that is, negative symptoms of diminished expression are key predictors of cognitive abilities in schizophrenia. Namely, communication and motor impoverishments predicted lower scores on tests of verbal memory, learning, visual attention, and working memory. These findings may inform personalized treatment approaches targeting cognitive deficits and negative symptoms in schizophrenia.

## Plain Language Summary Title

How speech and movement difficulties in schizophrenia link to specific thinking skills: insights from a study in Canada and Australia

## Plain Language Summary

What was the purpose of this study? We wanted to find out whether specific symptoms of schizophrenia, especially those related to speech, movement and gestures, are linked to specific thinking problems, such as verbal memory and learning or attention and working memory difficulties. Why does this matter? Schizophrenia affects how people think, feel, and behave. It includes two main types of symptoms: Positive symptoms: added experiences like hearing voices or unusual beliefs. Negative symptoms: reduced abilities, such as speaking less, moving less, showing fewer facial expressions, or withdrawing socially. Cognitive problems are common in schizophrenia, but it is unclear how they relate to these symptoms. Understanding this could improve treatment. What did we do? We studied 198 people with schizophrenia from Canada and Australia. They completed thinking tests and were assessed for a wide range of symptoms. What did we find? We found that only negative symptoms were related to cognitive difficulties. Specifically: People with more trouble expressing themselves verbally did worse on memory and verbal learning tasks. People with reduced movements and gestures did worse on attention and working memory tasks that required quick hand responses. Why is this important? Different negative symptoms affect different areas of thinking. This means treatments could be designed to target both the symptoms and the specific thinking problems that come with them. Take-away message: Looking at individual symptoms, like speech difficulties or reduced movement, offers clearer insight into specific thinking challenges in schizophrenia than relying on overall symptom scores. This could lead to more targeted and effective care.

## Résumé

**Objectif:**

Les troubles cognitifs sont une caractéristique fondamentale des troubles du spectre de la schizophrénie. Notre étude précédente portant sur une cohorte de participants ayant présenté un premier épisode psychotique a montré que les symptômes liés à la communication appauvrie ou désorganisée et aux déficits moteurs étaient respectivement des facteurs prédicteurs des scores liés à la mémoire verbale et à la mémoire de travail. Cette étude visait à évaluer ces facteurs prédicteurs chez des personnes présentant différents degrés de chronicité de la maladie.

**Méthodologie:**

Nous avons fait appel à une analyse en composantes principales itérative sous contraintes (iCPCA, de l’anglais *iterative Constrained Principal Component Analysis*) pour évaluer le lien entre 15 mesures cognitives tirées de la batterie de tests MATRICS *Consensus Cognitive Battery* (MCCB), y compris la vitesse de traitement, l’attention, la mémoire de travail, la mémoire verbale et la mémoire non verbale, le raisonnement ainsi que la résolution de problèmes, et 27 éléments de l’échelle PANSS (*Positive and Negative Syndrome Scale*). L’étude a été menée auprès de 198 patients en consultation externe provenant de deux centres situés en Australie et au Canada. La méthode iCPCA a été utilisée pour déterminer les symptômes qui permettent de prédire de façon fiable des combinaisons précises de mesures cognitives tout en contrôlant les erreurs de type 1.

**Résultats:**

Nous avons déterminé que l’élément *Manque de spontanéité et de fluidité de la conversation* de l’échelle PANSS permettait de prédire la composante liée à la mémoire verbale et à l’apprentissage, et que l’élément *ralentissement psychomoteur* de l’échelle PANSS était lié à la composante d’attention visuelle et de mémoire de travail.

**Conclusions:**

Ces résultats concordent avec nos constatations antérieures liées à un échantillon constitué de personnes ayant présenté un premier épisode psychotique, c’est-à-dire que les symptômes négatifs d’un déficit d’expression sont des facteurs prédicteurs importants des capacités cognitives chez les personnes atteintes de schizophrénie. Plus précisément, l’appauvrissement de la communication et les déficits moteurs étaient des facteurs prédicteurs de scores inférieurs aux tests portant sur la mémoire verbale, l’apprentissage, l’attention visuelle et la mémoire de travail. Ces résultats pourraient permettre d’orienter des approches de traitements personnalisées ciblant les déficits cognitifs et les symptômes négatifs chez les personnes atteintes de schizophrénie.

## Introduction

Cognitive impairment (CI) is a prominent feature of schizophrenia spectrum disorders (SSD). Cognitive impairment is pervasive (seen in around 80% of patients with SSD), presents early, persists throughout the illness, and predicts functional outcomes.^1^ Cognitive impairment has been observed across all core cognitive processing domains, significantly affecting processing speed, working memory, and verbal memory.^2,3^ The most promising therapeutic interventions are psychosocial interventions, particularly cognitive remediation training (CRT).^4–6^

Meta-analyses also showed that CRT has an impact on positive and negative symptoms.^4,5^ Similarly, numerous studies have shown general associations between symptom clusters and domains of cognition.^7–10^ However, understanding the nuanced relationship between combinations of individual symptoms (rather than just “positive” or “negative” symptoms) and specific aspects of cognition could lead to a deeper understanding of the pathophysiology of symptoms, and potentially lead to new treatment targets to improve symptoms and cognition.

Many studies examining the relationship between symptoms and cognition in people with SSDs have demonstrated that negative symptoms and disorganization, but not positive symptoms, are associated with CI, particularly in the domains of working and verbal memory.^9–18^ However, most of these studies relied on summary score methodologies, using aggregate scores for positive and negative symptoms or their subscales, and relating them to summary scores of cognitive deficits.^12,16^ This makes it difficult to determine which combinations of individual symptom items are the optimized predictors of specific combinations of cognitive scores.

In our previous work,^7,19^ we developed iterative Constrained Principal Component Analysis (iCPCA), a method for determining which combinations of individual symptom items are the optimized predictors of specific combinations of cognitive scores. This approach combines multivariate multiple regression and principal component analysis (PCA) into a unified framework. A key feature of iCPCA is that the resulting components indicate how strongly each measure contributes to a given dimension, providing a nuanced picture of symptom–cognition relationships. This stands in contrast to the common but overly simplistic practice of relying on aggregate or total scores, for instance, summing symptom items or cognitive test scores into global indexes, which risks obscuring important item-level patterns. In the current analysis, we extracted components from a set of cognitive measures optimized for their relationship with individual symptoms.

In a sample of first-episode psychosis patients FEP; detailed in reference,^12^ we observed two components of cognitive functions, Verbal Memory (C1) and Working Memory (C2), that were optimally predicted by symptoms derived from the Scale for the Assessment of Negative Symptoms (SANS)^20^; and the Scale for the Assessment of Positive Symptoms (SAPS).^21^ Symptoms related to impoverished (SANS symptom *Increased Latency of Responses*) and disorganized (SAPS symptom *Illogicality*) communication, language, and thought overlapped with the Verbal Memory component. Symptoms of motor impoverishment (from SANS: *Paucity of Expressive Gestures* and *Affective Nonresponsivity*) overlapped with the Working Memory component.

In the study reported here, we conducted a parallel analysis of the relationship between symptoms and cognition in a sample of outpatients diagnosed with schizophrenia from three different sites in Australia and Canada. The participants had varying illness durations, with the vast majority being chronic (more than 2 years from the onset of the symptoms). Psychotic symptoms were measured using the Positive and Negative Syndrome Scale (PANSS)^22^; and cognition using the Measurement and Treatment Research to Improve Cognition in Schizophrenia (MATRICS) Consensus Cognitive Battery (MCCB).^23,24^

This analysis was conducted to extend our previous results obtained with the first episode sample^7^ in an independent sample. Accordingly, we expected that two components of cognition, Verbal Memory and Working Memory, would be most strongly predicted by certain negative and disorganization symptoms. Specifically, symptoms related to impoverished and disorganized thinking, language, and communication would predict Verbal Memory scores, whereas symptoms of motor impoverishment would predict Working Memory scores.

## Methods

### Participants

The data used in this study were from previously reported patient data (*n* = 198 [126 males]; mean age = 41.26, *SD* = 10.84) collected in Canada and Australia. The 54 Canadian participants were tested at St. Joseph's Healthcare Hamilton and the Hamilton Program for Schizophrenia in Hamilton, Ontario, Canada. Participants were outpatients diagnosed with schizophrenia; only four of them were within 2 years from symptom onset (2% of the total sample), and the rest were chronic. Details of recruitment processes and ethical approvals are described elsewhere.^25,26^ Data from 88 participants (Australia I) were obtained from the Cognitive and Genetic Explanations of Mental Illnesses biodata bank for details.^27,28^ Finally, 56 participants (Australia II) were from a more extensive data set of participants recruited from public hospitals and outpatient facilities in the Australian cities of Melbourne, Brisbane, Adelaide, and Sydney as part of a clinical trial of N-acetyl cysteine for treatment-resistant schizophrenia.^29,30^ All participants in both sites in Australia had a chronic illness. Table 1 shows a comparison of demographic and summary symptom statistics across groups.

**Table 1.** The Three Samples’ Demographic and PANSS Summary Characteristics, with F and *P* Values from one-way ANOVA.

Variable	Statistic	Canada	Australia I	Australia II	Total	χ² or *F*	*P* value
Sex Females:Males	N	18:36	37:51	17:39	72:126	2.31	0.31
Symptoms onset (years)	Mean	17.75	22.95	23.84	21.60	**5**.**17**	**<0**.**01**
	SD	11.98	9.78	7.22	10.16		
	N	46	58	44	148		
Education years	Mean	13.15	14.07	12.96	13.5	2.97	0.05
	SD	3.08	3.25	1.85	2.89		
	N	52	81	55	188		
Age years	Mean	38.59	42.44	41.98	41.26	2.32	0.10
	SD	9.23	11.17	11.46	10.84		
	N	54	88	56	198		
PANSS positive	Mean	17.62	15.71	15.73	16.23	2.33	0.10
	SD	6.5	5.13	4.72	5.46		
	N	52	85	55	192		
PANSS negative	Mean	21.7	14.86	15.43	16.89	**26**.**50**	**<0**.**01**
	SD	5.38	6.32	4.97	6.41		
	N	54	88	56	198		
PANSS general	Mean	36.39	30.24	30.57	32.01	**11**.**80**	**0**.**01**
	SD	7.46	8.57	6.68	8.19		
	N	54	88	56	198		

*Note*. Chi-square (χ²) analysis was used only for the sex comparison among the three sites. PANSS = Positive and Negative Syndrome Scale.

### Cognitive Measures: the MCCB

The MCCB is a set of tests that measure seven main domains of cognition: speed of processing, attention/vigilance, working memory, verbal learning, visual learning, reasoning and problem-solving, and social cognition in schizophrenia.^23,24^ Not all MCCB tests were administered in all three research centers, so we selected the tests with the most completed entries; in total, there were 15 cognitive variables (Table 2). Please refer to Table S1 in Supplementary Material for site-wise comparisons of cognitive scores.

**Table 2.** MATRICS Cognitive Measures Included in the iCPCA.

Cognitive domain	Test	Description	Variable analyzed
Processing speed	Trail Making Test Part A (TMT)	Participants connect dots arranged in a pattern as quickly as possible.	Time to complete the task (seconds).
Processing speed	Digit Symbol Coding (DSC)	Measures the time taken to write digits corresponding to nonsense symbols.	Number of correct symbols completed.
Sustained attention	Continuous Performance Task Identical Pairs (CPT-IP): 2-digits, 3-digits, and 4-digits.	Participants press a button when presented with matching numbers across three conditions (2-digits, 3-digits, 4-digits).	*d′* measure of accuracy for each of the three trials.
Working memory (Nonverbal)	Spatial Span (SS): backward and forward	Participants arrange cubes in the same and reverse sequence as demonstrated by the test administrator.	Total correct sequences (forward and backward).
Working memory (Verbal)	Letter-Number Sequence (LNS)	Participants repeat aloud a sequence of letters and numbers heard from the test administrator.	Total correct sequences.
Verbal memory and learning	Hopkins Verbal Learning Task (HVLT)	Participants recall 12 words immediately in three trials.	Number of correct words recalled
Non-Verbal memory and learning	Brief Visuospatial Memory Test-Revised (BVMTR)	Consists of three trials measuring immediate recall of geometric figures.	Number of correct figures recalled.
Reasoning and problem-solving	Mazes	Participants use foresight and planning to navigate through mazes of increasing difficulty.	Total number of mazes correctly completed.

iCPCA = iterative Constrained Principal Component Analysis.

### Symptom Rating Scales: Positive and Negative Syndrome Scale

Positive and Negative Syndrome Scale consists of 30 items measuring three symptom categories: positive, negative, and general.^22^ Clinical interviewers rate the severity of each symptom using a 7-point scale (from 1—absent to 7—extremely severe).

To create a set of symptoms that parallels those used in our previous study, we eliminated symptoms that essentially measure cognitive performance^7^; namely, those items which fall into the cognitive factor of the PANSS^31^: Difficulty in Abstract Thinking (N5), Poor Attention (G11), and Disorientation (G10). The final 27 symptom items are listed in Table 3, and the PANSS summary scores are presented in Table 1.

**Table 3.** Mean Predictor Loadings from the PANSS for the Predicted Solution in Two Components.

PANSS items	C1	C2
P1 Delusions	0.02	0.22
P2 Conceptual disorganization	−0.06	−0.34
P3 Hallucinations	−0.21	0.10
P4 Excitement	−0.04	0.02
P5 Grandiosity	−0.03	0.26
P6 Suspiciousness	0.05	0.12
P7 Hostility	−0.04	−0.16
N1 Blunted affect	−0.29	−0.05
N2 Emotional withdrawal	−0.20	−0.03
N3 Poor rapport	−0.23	0.03
N4 Social withdrawal	−0.01	−0.15
N6 Lack flow conversation	**−0**.45**	−0.14
N7 Stereotyped thinking	−0.14	−0.13
G1 Somatic concern	−0.07	0.01
G2 Anxiety	0.36	−0.02
G3 Guilt	0.16	0.04
G4 Tension	0.10	0.07
G5 Mannerisms	−0.33	−0.09
G6 Depression	0.16	−0.04
G7 Motor retardation	−0.16	**−0**.40*
G8 Uncooperativeness	−0.06	0.02
G9 Unusual thought	−0.04	0.27
G12 Lack of insight	0.04	0.00
G13 Disturb volition	−0.03	−0.15
G14 Poor impulse control	0.03	−0.13
G15 Preoccupation	−0.10	−0.08
G16 Social avoidance	0.00	−0.02

*Note*. The values are Person *r* coefficients. Significant predictor loadings are set in bold font. * *P* ≤ 0.05, ** *P* ≤ 0.01 from the Benjamini–Hochberg multiple comparison correction test. PANSS = Positive and Negative Syndrome Scale.

### Data Analysis

#### Iterative Constrained PCA

Constrained PCA (CPCA) is a supervised dimensionality reduction technique that combines multivariate multiple regression's variance constraints and PCA's dimensionality reduction into a unified framework.^32,33^ In other words, the CPCA technique relies on a specific set of predictor variables to guide the dimensionality reduction process. Constrained PCA provides a set of component and predictor loadings that link the low-dimensional component scores to the original criterion and predictor variables, respectively.

In the current study, we used the iCPCA^7^ to determine consistency and reliability in the optimized combination of items overlapping between criterion and predictor variables. More specifically, to assess the reliability of the predictor loadings across all the iterations, we used a metric termed the predictor loading reliability proportion (*PLRP*). This metric is computed as the proportion of iterations (here expressed as a percentage) that returned predictor loadings above a certain threshold (Pearson's *r* = 0.26) in both split-half solutions for 1,000 random permutations. The supplementary material details our methods for selecting the optimal threshold value. We only interpreted *PLRP* values that passed the *P* < 0.05 after correcting for multiple comparisons using the Benjamini–Hochberg correction.^34^

Predictor loadings and component loadings both offer essential but distinct pieces of information about the same components in a CPCA, so they must be interpreted together. Component loadings demonstrate the relative importance of each criterion variable (i.e., cognitive measure) for each component, whereas predictor loadings show how each predictor variable (i.e., symptom) relates to each cognitive component. More specifically, component loadings are calculated by correlating the component scores with the variance-constrained criterion variables (cognitive measures), while predictor loadings are obtained by correlating the component scores with the predictor variables (symptoms). We determined how many cognitive measures (component loadings) to retain for each component by using the average *PLRPs*, which involve regressing each criterion variable on the remaining variables. Additional details are provided in Figure S4 of Supplementary Material. Lastly, iCPCA component scores were also employed to examine relationships with other factors, such as age and years of education.

#### Software and Scripts

The data were analyzed using MATLAB (The MathWorks, Natick, MA) and IBM SPSS (IBM Corp. Released 2021. IBM SPSS Statistics for Windows, Version 28.0. Armonk, NY: IBM Corp). The iCPCA MATLAB script is available on our GitHub website: https://github.com/CNoS-Lab/iCPCA.

## Results

### The Comparison of Demographic and Summary PANSS Scores among Three Sites

Table 1 details demographic and summary PANSS scores of the samples. Importantly, ANOVA revealed no significant differences years of education (*F*(2,185) = 2.97, *P* = 0.05, *η^2^* = .03), age (*F*(2,195) = 2.32, *P* = 0.101, *η^2^* = .02), or PANSS Positive (*F*(2,189) = 2.33, *P* = 0.10, *η^2^* = .02). The difference among the three sites was significant for the PANSS Negative, *F*(2,195) = 26.50, *P* < 0.001, *η^2^* = .21, indicating a large effect size. The Scheffé post hoc comparisons showed significant mean differences between Canada and Australia II (*M* = 6.28*, P* < 0.01). Additionally, there was a significant difference in the PANSS General, *F*(2,195) = 11.80, *P* < 0.01, *η^2^* = .11, indicating a moderate effect size. The post hoc comparisons revealed significant mean differences between Canada and Australia II (*M* = 5.82, *P* = 0.001) and between Canada and Australia I (*M* = 6.15, *P* < 0.01). Finally, no significant sex differences were observed among the three sites, as measured by a chi-square test of independence, χ²(2, *N* = 198) = 2.31, *P* = 0.31.

When combining data from all three sites, there was a significant difference in years of education between males (*M* = 13.15, *SD* = 2.84) and females (*M* = 14.07, *SD* = 2.90); *t*(186) = −2.15, *P* < 0.05, *d* = 0.32). There were no other significant differences between males and females for the demographic and clinical variables.

### The iCPCA Results

The multivariate overlap between cognitive measures and symptom rating scale items was 30.76%, averaged across all 1,000 iterations. We extracted two components from PCA, determined by the scree plot^35^ and Horn's parallel analysis.^36^ The scree plot is shown in Figure S3 in Supplementary Material. The components were varimax rotated. Specific predictor loadings significantly predicted these two components, which accounted for 60.4% of the symptom-constrained cognitive score variance averaged over all iterations (C1 = 31.3%, C2 = 29.1%).

**Figure 1.**
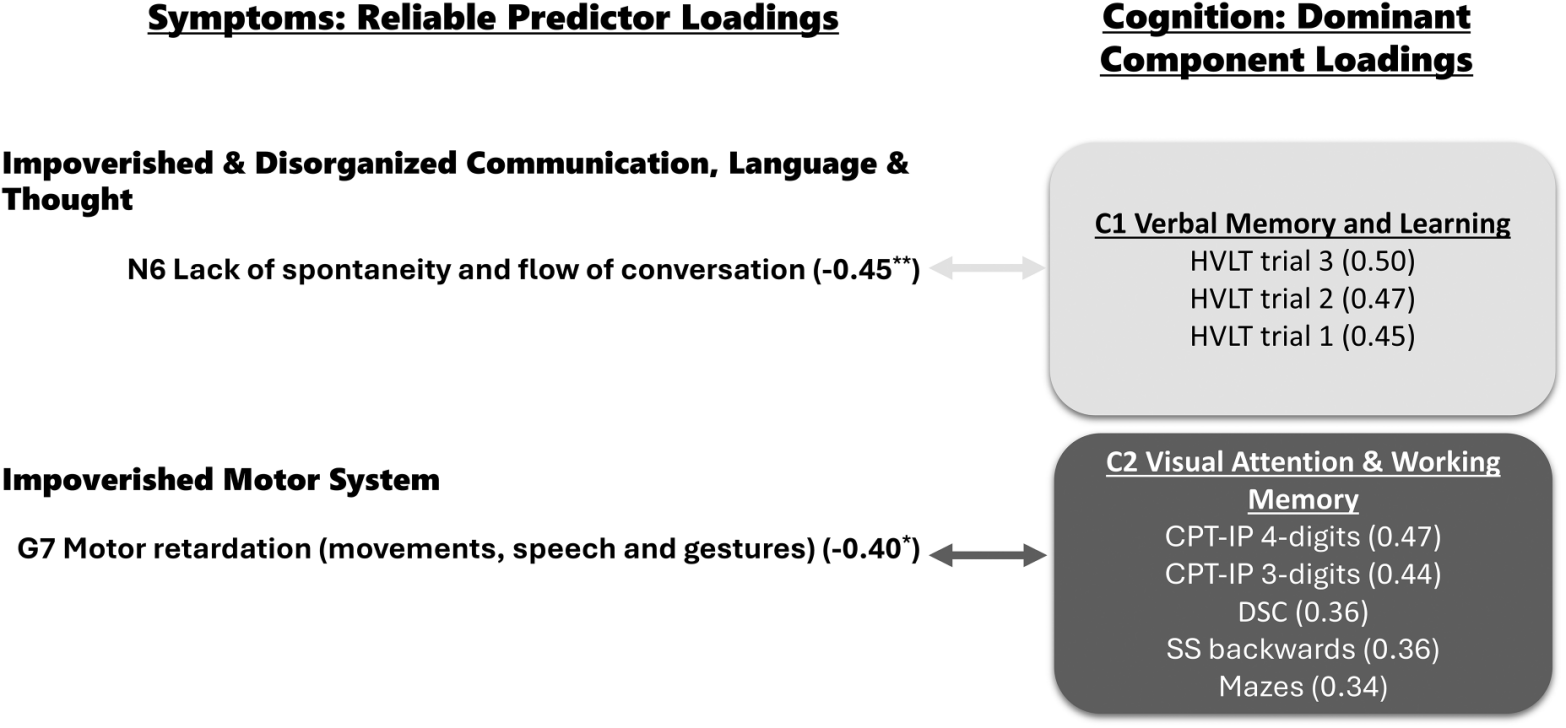
On the left side, are reliable predictor loadings grouped into symptom types. On the right side are components (C) and their dominant component loadings grouped by two colors: light blue is C1, and dark pink is C2. The light and dark arrows connect symptoms with C1 and C2, respectively. The component and predictor loading values (in parenthesis) measure effect size; a minus sign precedes negative values. * *P* ≤ 0.05, ** *P* ≤ 0.01 from the Benjamini–Hochberg multiple comparison correction test. CPT-IP = Continuous Performance Task Identical Pairs; DSC = Digit Symbol Coding; HVLT = Hopkins Verbal Learning Task; SS = Spatial Span.

We selected three component loadings for C1 and four for C2, based on Figure S4 in Supplementary Material. The figure illustrates the selection process for component loadings based on the average predictor reliability proportion.^7^ Figure 1 summarizes the method of component interpretation, highlighting the selected criterion variables and their associated reliable predictor variables for each component. Additionally, Table 4 lists all 15 component loadings, and Table 3 all 27 predictor loadings.

**Table 4.** Component Loadings for the Predicted Solution.

**MATRICS test**	**C1**	**C2**
TMT Part A	−0.28	−0.20
DCS	0.26	**0**.**36**
CPT-IP 2-digit	0.12	0.39
CPT-IP 3-digit	0.12	**0**.**44**
CPT-IP 4-digit	0.12	**0**.**47**
SS forward	0.14	0.30
SS backwards	0.13	**0**.**36**
LNS	0.28	0.33
HVLT trial 1	**0**.**45**	0.18
HVLT trial 2	**0**.**47**	0.15
HVLT trial 3	**0**.**50**	0.22
BVMTR trial 1	0.35	0.09
BVMTR trial 2	0.39	0.12
BVMTR trial 3	0.39	0.19
Mazes	0.19	**0**.**34**

*Note.* Dominant component loadings are set in bold font. Figure S4 specifies the selection of the component loadings based on the average predictor reliability proportion. BVMTR = Brief Visuospatial Memory Test-Revised; CPT-IP = Continuous Performance Task Identical Pairs; DSC = Digit Symbol Coding; HVLT = Hopkins Verbal Learning Task; LNS = Letter-Number Sequence; SS = Spatial Span; TMT = Trail Making Test.

Component 1 (C1) was dominated by three variables: *HVLT immediate recall trial 3* (*r* = −0.50), *HVLT immediate recall trial 2* (*r* = −0.47), and *HVLT immediate recall trial 1* (*r* = −0.45). The three *HVLT* test trials assess verbal memory and learning over the course of three trials, and short-term/verbal working (see Table 2). We labeled this component as C1 Verbal Memory and Learning. This component overlapped significantly with one symptom: *Lack of Spontaneity and Flow of Conversation* (PANSS N6); *r* = −0.45, *PLRP* = 96%, *P* < 0.01.

Component 2 (C2) consisted of attention, processing speed, and working memory measures: *CPT-IP 4-digits* (*r* = 0.47) and *3-digits* (*r* = 0.44), *DSC* (*r* = 0.36), *SS backward* (*r* = 0.36), and *Mazes* (*r* = 0.34). The tasks are described in detail in Table 2. We labeled this component as C2 Visual Attention and Working Memory. C2 overlapped with one symptom: *Motor Retardation* (PANSS G7) (*r* = −0.40, 81%, *P* < 0*.*05).

### Relationship of iCPCA Components to Other Measures and Their Comparison Among the Sites

A comparison of the component scores (averaged over 1,000 iterations) revealed that for Attention and Working Memory (C2), there was a significant difference between males (*M* = 0.06, *SD* = 0.77) and females (*M* = −0.24, *SD* = 0.87); *t*(196) = 2.48, *P* < 0.01, *d* = 0.37). Males had higher scores on the Attention and Working Memory component, with a small to medium effect size.

A Pearson correlation analysis examined the relationships between age, years of education, and two cognitive components: Verbal Memory and Learning (C1) and Visual Attention and Working Memory (C2). There was a significant, yet weak correlation between age and C1, *r*(198) = −0.18, *P* < 0.01. There were no more significant correlations between age and Attention and Verbal Memory, *r*(198) = −0.11, *P* = 0.13, between age and years of education, *r*(198) = 0.08, *P* = 0.25, between years of education and C1, *r*(188) = 0.13, *P* = 0.07, and C2, *r*(188) = 0.06*, P* = 0.40.

We also compared the component scores across three sites. The ANOVA revealed significant differences in the case of Verbal Memory and Learning (C1); *F*(2, 195) = 4.17, *P* = 0.02, *η^2^* = .04, indicating small effect size. The Scheffé post hoc comparisons showed significant mean differences between Australia I and Australia II (*M* = −0.40*, P* = 0.02). There were no significant differences in the case of Visual Attention and Working Memory C2; *F*(2, 195) = 1.98, *P* = 0.14, *η^2^* = .02.

## Discussion

Our study confirmed that two cognitive components, Verbal Memory and Learning (C1) and Visual Attention and Working Memory (C2), are associated with distinct symptom clusters in people with chronic schizophrenia. C1 was best predicted by the PANSS item *Lack of Spontaneity and Flow of Conversation*, reflecting impoverished verbal communication. C2 was predicted by *Motor Retardation*, associated with expressive and motor behavior. These findings extend our previous results in our FEP sample.^7^

When comparing the current findings to our previous study, it is important to note that the two samples differed in illness stage: FEP in the earlier study and mostly chronic schizophrenia in the current one. Both groups consisted of outpatients, but the chronic sample was older and had more years of education, likely reflecting age-related factors. Symptom profiles also differed; FEP participants exhibited more prominent negative symptoms and fewer positive or disorganization symptoms, while the chronic group showed moderate levels across domains. This pattern may reflect a sampling bias, as patients with persistent symptoms tend to remain in specialist care. Despite these differences, prior research suggests more similarities than differences in symptom manifestation between FEP and chronic schizophrenia patients.^37,38^

Although the two studies used different cognitive batteries and symptom rating scales, the assessed domains were largely comparable. Both studies evaluated key areas of cognitive function, including working memory, verbal memory, processing speed, attention, and executive functioning.^7,12^ However, there were some differences. The three trials of the HVLT used here assess immediate word learning and do not include recognition or delayed recall components, unlike the Logical Memory subtest from the Wechsler Memory Scale used in our previous study. This is why we renamed C1 to emphasize the learning aspect of the HVLT. Furthermore, all attention tests that loaded onto C2 were based on visual stimuli with speeded motor responses, and we chose a new name for this component to reflect that as well.

Similarly, while the earlier study employed SANS and SAPS to provide detailed ratings of negative and positive symptoms, the current study used PANSS, which aggregates symptom domains more broadly and is commonly used in clinical trials.^22^ Despite structural differences, strong correlations exist between these instruments.^39,40^ For example, the PANSS item *Lack of Spontaneity and Flow of Conversation* captures core aspects of alogia assessed across multiple items in SANS,^20,41^ and *Motor Retardation* reflects both motor and communicative features found in SANS's Affective Flattening subscale.^22^ Thus, despite using different tools, both studies identified similar symptom patterns predicting cognitive abilities. Notably, in both cases, negative symptoms related to diminished expression, not apathy, were consistently associated with verbal and working memory.^42^

A cognitive model of diminished expressions proposed by García-Mieres and colleagues^43^ states that diminished expressions are related to three interacting processes: cognitive symptoms, impaired self-reflectivity, and higher-order language disturbances. Our current and previous results support this model's first and last domains. The overlap between impoverished communication, verbal memory, and learning seen in C1 could be related to a lack of cognitive resources needed to perform verbal memory tasks.^44^ This process may result from the disorganization of semantic knowledge,^45,46^ which is crucial for verbal memory^47^ measured by the *HVLT*, thereby preventing efficient access to memories.^48^ The relationship between motor impoverishment and attention and working memory observed on C2 also supports the cognitive model of diminished expression. It points to a disturbed interaction between the motor system governing non-verbal communication and cognitive functions.^49^ This process is often seen in SSD patients with reduced gesture use and gesture control.^49–51^

The two components observed in both studies may implicate distinct brain networks involved in the severity of diminished expression. In the case of C1, this may be the language network.^52,53^ This brain system has been previously defined as a crucial biomarker of the severity of language and communication disturbances in SSD.^54–56^ In the case of C2, there are prefrontal mechanisms crucial for performance in working memory tasks, such as the dorsolateral prefrontal cortex and the anterior cingulate cortex, which are often impaired in the SSD population.^57,58^ Other working-memory-related brain regions that may be implicated in this overlap are cortical premotor and motor areas, cerebellum, thalamus, and insufficient interaction of thalamocortical loops.^59–63^ A bilateral network of mainly frontoparietal brain areas is engaged during the planning, execution, perception, and interpretation of gestures.^64^ Future studies should investigate the effectiveness of neurostimulation protocols for the brain mechanisms involved in these two components, which may reduce the cognitive burden and elevate negative symptoms.^65^

Turning to potential therapeutic implications of our findings, further development of specialized psychotherapies, such as CRT targeting specific symptoms, may be beneficial in improving cognition and reducing the impact of negative symptoms on patients’ lives via cognitive mechanisms. Given the consistent link between expressive symptoms and verbal memory deficits, CRT interventions that emphasize verbalization strategies may be particularly effective in improving functional outcomes among patients with diminished expression.^66,67^ There are also emerging forms of psychotherapy focused on a metacognitive understanding of particular skills, such as social communication, showing benefits in reducing the severity of negative symptoms.^68^ Furthermore, metacognitive training for psychosis,^69^ traditionally associated with the alleviation of positive symptoms, also helps mitigate the burden of negative symptoms.^70^

This study allowed us to overcome some of the limitations of our previous analysis^7^; namely, increased sample size and a roughly equal positive and negative symptoms ratio. However, symptom measures were collected across three different sites, which prevent us from uniformly equating the assignment of each interviewer across different centers. As shown in Table 1, there were differences in symptom severity, particularly regarding negative symptoms, between the sites. Additionally, differences were observed in MCCB results and component scores, especially for C1 (Verbal Memory and Learning). Participants from Australia II displayed the lowest cognitive abilities (and education level), particularly evident in the *HVLT* trials (refer to Tables S1 and S2). Therefore, while this merged analysis is not ideal, it still provides an important basis for further studies. In the future, it would be important to verify the relationship between symptoms and cognition in more standardized cross-site designs, ideally utilizing diverse languages and cultural settings. This approach could enhance cross-site harmonization and the validity of our findings based on the iCPCA methodology. It is also important to note that neither study used apathy- or motivation-specific questionnaires. It would be beneficial for future iCPCA studies to examine the role of apathy in CI using symptoms measured by tools specifically designed to assess apathy and motivation in schizophrenia. Lastly, both of our studies showed that motor-related symptoms influence performance on cognitive tasks, which are proxies for cognitive domains and largely rely on speeded responses. Future studies should consider using working memory tests that rely less on motor responses to better disentangle the role of motor slowness in cognitive performance.

In conclusion, the iCPCA conducted on a group of chronic patients with schizophrenia revealed that symptoms related to impaired and disorganized communication, language, and thought predicted reduced verbal memory and learning, while symptoms related to motor impairment predicted visual attention and working memory. Importantly, there was no association between cognitive abilities and positive symptoms. These findings link negative symptoms of diminished expression to cognitive function. Consequently, our results should encourage further data-driven dimensional research into schizophrenia defined by combinations of individual symptoms and cognitive profiles. Furthermore, such efforts may inform psychotherapy studies aimed at targeting the specific symptoms related to CI in schizophrenia.

## Supplemental Material

sj-docx-1-cpa-10.1177_07067437251387565 - Supplemental material for A multisite study of the overlap between symptoms and cognition in schizophrenia: Une étude multicentrique sur le chevauchement entre les symptômes et les troubles cognitifs chez les personnes atteintes de schizophrénie Supplemental material, sj-docx-1-cpa-10.1177_07067437251387565 for A multisite study of the overlap between symptoms and cognition in schizophrenia: Une étude multicentrique sur le chevauchement entre les symptômes et les troubles cognitifs chez les personnes atteintes de schizophrénie by Rafal M. Skiba, Abhijit M. Chinchani, Mahesh Menon, Martin Lepage, Katie M. Lavigne, Ashok Malla, Ridha Joober, Joel O. Goldberg, R. Walter Heinrichs, David J. Castle, Amy Burns, Michael W. Best, Susan L. Rossell, Sebastian Walther and Todd S. Woodward in The Canadian Journal of Psychiatry
