# Supplementary material for: A multisite study of the overlap between symptoms and cognition in schizophrenia: Une étude multicentrique sur le chevauchement entre les symptômes et les troubles cognitifs chez les personnes atteintes de schizophrénie
Source: Can J Psychiatry. 2025 Oct 22;71(3):199–209. doi: 10.1177/07067437251387565 (PMC12546101; doi:10.1177/07067437251387565)

**Supplementary Material:**

**A multi-site study of the overlap between individual differences in cognition and symptoms of schizophrenia**

Rafal M. Skiba^1,2^, Abhijit M. Chinchani^1,2,3^, Mahesh Menon^2^, Martin Lepage^4,5^, Katie M. Lavigne^4,5^, Ashok Malla^4,5^, Ridha Joober^4,5^, Joel O. Goldberg^6^, R. Walter Heinrichs^6^, David J Castle^7^, Amy Burns^2^, Michael W. Best^8^, Susan L. Rossell^9,10^, Sebastian Walther^11,12^, and Todd S. Woodward^1,2,*^

^1^BC Mental Health and Addictions Research Institute, Vancouver, BC, Canada

^2^Department of Psychiatry, University of British Columbia, Vancouver, BC, Canada

^3^Department of Bioinformatics, University of British Columbia, Vancouver, Canada

^4^Douglas Research Centre, McGill University, Montreal, QC, Canada

^5^Department of Psychiatry, McGill University, Montreal, QC, Canada

^6^Department of Psychology, York University, Toronto, ON, Canada

^7^Department of Psychiatry, University of Tasmania, Hobart, TAS, Australia

^8^Department of Psychological Clinical Science, University of Toronto, Toronto, ON, Canada

^9^Centre for Mental Health, Swinburne University of Technology, Melbourne, VIC, Australia

^10^Department of Mental Health, St Vincent’s Hospital, Melbourne, VIC, Australia

^11^Translational Research Center, University Hospital of Psychiatry and Psychotherapy, University of Bern, Switzerland

^12^Department of Psychiatry, Psychosomatics, and Psychotherapy, Center of Mental Health, University Hospital Würzburg, Germany

* Corresponding Author. Please address all correspondence to: Todd S. Woodward, Ph. D., Room A3-A116, BC Mental Health & Addictions Research Institute – Translational Research Building, 3rd Floor, 938 W. 28th Avenue, Vancouver, British Columbia, Canada, V5Z 4H4, fax: 604-875-3871, phone: 604-875-2000 x 4724, e-mail: [Todd.S.Woodward@gmail.com](about:blank).

**1. CPCA Methods**

Constrained Principal Component Analysis (CPCA) is a supervised dimensionality reduction method that integrates the variance constraints of multivariate multiple regression with the dimensionality reduction of PCA within a single framework. In the present application, CPCA is used to extract orthogonal dimensions from the variance in the criterion variables specifically, 15 cognitive measures (from MATRICS) that are optimized to align with a set of predictor variables (27 items from the Positive and Negative Syndrome Scale). CPCA consists of two primary steps: external and internal analyses. In the external analysis, multivariate multiple regression is employed to partition the total variance in the criterion variables (denoted as **Z**) into two components: the variance that can be explained by the predictor variables (**G**) and the variance that cannot. This process decomposes the criterion variable matrix (**Z**) into two matrices: one containing the regression-based estimated scores (**GC**) and the other comprising the residual or error scores (**E**). The external analysis is represented by the standard regression equation:

$$Z = GC + E$$

Where *Z* = matrix of criterion variables, *G* = matrix of predictor variables, $C={(G'G)}^{-1}G'Z$ is a matrix of regression coefficients, *GC* = regression-based estimated score matrix, and *E* = residual scores (variance in *Z* not explained by predictor variables in *G*). This external analysis is followed up by the internal analysis, which involves performing a PCA (or singular value decomposition) on the predicted scores (*GC)*, and can be denoted as follows:

$$GC = UDV’$$

where *U* is the matrix of left singular vectors, and *V* is the matrix of right singular vectors. When *V* is combined with the diagonal matrix *D*, this gives the component loadings *(*$VD/\sqrt{n}$*)*, where *n* is the number of data points (i.e. participants in this case). Component loadings indicate the contribution of each criterion variable to the component. When the square root of *n* scales *U*, this provides the component scores *(*$\sqrt{n} U$*).* Component scores *(*$\sqrt{n} U$ *)* are further correlated with *G* columns to create predictor loadings *(P).* These predictor loadings indicate the degree to which each predictor relates to each component score extracted in the PCA of *GC.* The component loadings indicate the importance of each criterion variable (cognition) for each component, and the predictor loadings indicate the importance of each predictor variable (symptom rating item) for each component. Component loadings and predictor loadings must be interpreted in conjunction because they are different pieces of information about the same components.

### **1.1. Combining results from different iterations**

The CPCA analysis was conducted 2,000 times (2 halves × 1,000 iterations). A key challenge in performing dimensionality reduction repeatedly is that components from different CPCA runs cannot be directly compared. Specifically, predictor loadings from one iteration do not exactly align with those from another, as the derived components differ across iterations. To overcome this issue ^1^, we first vertically concatenate the *GC* matrices across all iterations to generate a *concatenated* *GC* matrix. The *concatenated GC* matrix has 1000*n (no. of iterations * no. of subjects) rows. Next, we perform only one PCA analysis on the concatenated *GC* matrix ^2^. Such an approach ensures that the component loadings for all folds/iterations are matched. Then, the computed concatenated component scores matrix is split into component scores for each fold/iteration, and the predictor loadings are computed for each fold/iteration separately. The mean predictor loadings across all the folds/iterations, and the component loadings from the concatenated *GC* matrix are used to interpret the results.

### **1.2. iCPCA in detail**

This section describes the iCPCA methodology in detail (also see Figure S1). The entire data of criterion variables ($\text{Z}_{n X zDim}$) and predictor variables ($\text{G}_{n X gDim}$) are divided into two halves: (${\text{(Z}\text{1}\text{)}}_{n/2 X zDim}$*,* ${\text{(G}\text{1}\text{)}}_{n/2 X gDim}$) and (${\text{(Z}\text{2}\text{)}}_{n/2 X zDim}$*,* ${\text{(G}\text{2}\text{)}}_{n/2 X gDim}$), where, *n* is the number of participants (213 in this case), *zDim* is the number of criterion variables (18 cognitive measures in this case), and *gDim* is the number of predictor variables (46 SANS+SAPS questionnaire items in this case).

CPCA is run separately on each of the split halves to get the *GC* matrix for each of the halves.

First half:

$${\text{(Z}\text{1}\text{)}}_{n/2 X zDim}\text{= }{\text{(G}\text{1}\text{)}}_{n/2 X gDim}{\text{(C}\text{1}\text{)}}_{gDim X zDim}\text{ + }{\text{(E}\text{1}\text{)}}_{n/2 X zDim}$$

Where,${\text{(C}\text{1}\text{)}}_{gDim X zDim}={(\text{G}\text{1}'\text{G}\text{1})}^{-1}\text{G}\text{1}'\text{Z}\text{1}$

Second half:

$${\text{(Z}\text{2}\text{)}}_{n/2 X zDim}\text{= }{\text{(G}\text{2}\text{)}}_{n/2 X gDim}{\text{(C}\text{2}\text{)}}_{gDim X zDim}\text{ + }{\text{(E}\text{2}\text{)}}_{n/2 X zDim}$$

Where,${\text{(C}\text{2}\text{)}}_{gDim X zDim}={(\text{G}\text{2}'\text{G}\text{2})}^{-1}\text{G}\text{2}'\text{Z}\text{2}$

The above split half procedure is repeated for 1000 iterations, and the *GC* matrices are obtained for each half and each iteration (as shown in Figure S1A). The *GC* matrices for all halves and iterations are concatenated along the rows to form a concatenated *GC* matrix (see section on **1.1.** **Combining results from different iterations** and Figure S1B).

$$\text{(GC)}_{1000n X zDim}\text{= }\left[ \begin{matrix} {(G_{1}C_{1})}_{n/2 X zDim}^{iteration 1} \\ {(G_{2}C_{2})}_{n/2 X zDim}^{iteration 1} \\ {(G_{1}C_{1})}_{n/2 X zDim}^{iteration 2} \\ {(G_{2}C_{2})}_{n/2 X zDim}^{iteration 2} \\ \vdots\\ {(G_{1}C_{1})}_{n/2 X zDim}^{iteration 1000} \\ {(G_{2}C_{2})}_{n/2 X zDim}^{iteration 1000} \end{matrix} \right]$$

PCA is performed on the concatenated GC matrix to generate component scores for each half/ iteration, and one common set of component loadings.

$$\text{(GC)}_{1000n X zDim}= \text{(U)}_{1000n X k}\text{(D)}_{k X k}\text{(V')}_{k X zDim}$$

Where, *k* is the number of components selected. In the current study, 3 components were selected based on the scree plot ^3; 4^. The common component loadings ($\text{L}$) and the concatenated scores ($\text{S}$) are computed as follows:

$$\text{(L)}_{zDim X m}= {\frac{1}{\sqrt{1000n}}\text{(V)}}_{zDim X k}\text{(D)}_{k X k}$$

$$\text{(S)}_{1000n X k}= {\sqrt{1000n}\text{ (U)}}_{1000n X k}$$

The concatenated scores are split into component scores for each of the halves and iterations and are correlated with the *G* matrices to obtain predictor loadings (*P*) for each iteration and each half.

$$\left[ \begin{matrix} {(S_{1})}_{n/2 X k}^{iteration 1} \\ {(S_{2})}_{n/2 X k}^{iteration 1} \\ {(S_{1})}_{n/2 X k}^{iteration 2} \\ {(S_{2})}_{n/2 X k}^{iteration 2} \\ \vdots\\ {(S_{1})}_{n/2 X k}^{iteration 1000} \\ {(S_{2})}_{n/2 X k}^{iteration 1000} \end{matrix} \right]\text{= }\text{(S)}_{1000n X k}$$

$$\begin{matrix} {(P_{1})}_{gDim X k}^{iteration 1} & = & {(G_{1})}_{n/2 X gDim}^{iteration 1} \star{(S_{1})}_{n/2 X k}^{iteration 1} \\ {(P_{2})}_{gDim X k}^{iteration 1} & = & {(G_{2})}_{n/2 X gDim}^{iteration 1} \star{(S_{2})}_{n/2 X k}^{iteration 1} \\ {(P_{1})}_{gDim X k}^{iteration 2} & = & {(G_{1})}_{n/2 X gDim}^{iteration 2} \star{(S_{1})}_{n/2 X k}^{iteration 2} \\ {(P_{2})}_{gDim X k}^{iteration 2} & = & {(G_{2})}_{n/2 X gDim}^{iteration 2} \star{(S_{2})}_{n/2 X k}^{iteration 2} \\ \vdots& = & \vdots\\ {(P_{1})}_{gDim X k}^{iteration 1000} & = & {(G_{1})}_{n/2 X gDim}^{iteration 1000} \star{(S_{1})}_{n/2 X k}^{iteration 1000} \\ {(P_{2})}_{gDim X k}^{iteration 1000} & = & {(G_{2})}_{n/2 X gDim}^{iteration 1000} \star{(S_{2})}_{n/2 X k}^{iteration 1000} \end{matrix}$$

Where, the ‘$\star$’ operator indicates Pearson’s correlation.

The means of the predictor loadings (*mean P*, Figure S1B) averaged over all the split halves and iterations indicate the effect sizes. To determine reliable predictor loadings in each iteration, we check if predictor loadings in both split halves have a correlation coefficient greater than an optimized value which, in this case, was |r| ≥ 0.26 (see section **2.** **Significance test and optimization of the hyperparameter (*r*).** We represent the reliability of predictor loadings as reliability binary (RB) matrices, where reliable and unreliable predictor loadings are assigned a value of ‘1’ and ‘0’, respectively. To determine the reliability of the predictor loadings across all the iterations, we computed a metric - predictor loading reliability proportions (*PLRP*, Figure S1B) - which is an average of the RB matrices across all iterations. In other words, for the current study, PLRP was the proportion of iterations that showed Pearson correlation *r* ≥ .26 in both split-half solutions.

**2. Significance Test and Optimization of the Hyperparameter (r)**

The significance of the PLRP values was assessed using a permutation test with 1,000 random permutations. For each permutation, the rows of the G matrix were randomly shuffled, and the entire iCPCA analysis (as previously described) was performed using the original Z matrix and the permuted G matrix to generate a PLRP matrix. This process was repeated 1,000 times. It is important to distinguish this procedure from the 1,000 iterations of split-halves. In the permutation test, the 1,000 iterations are used to compute a PLRP matrix for each permutation of the G matrix, thereby constructing the null distribution. PLRP values obtained from the non-permuted iCPCA analysis were considered significant if they met the threshold of *p* ≤ .05 after applying the Benjamini-Hochberg correction for multiple comparisons with an FDR of .05 ^5^. Thus, we interpreted predictor loadings for which *PLRP* values were deemed significant in the permutation test.

The *PLRP* null distribution generated above varies as a function of the hyperparameter, *r*. The *PLRP* null distributions for three different *r*-values are shown in Figure S2B-D. For example, if the *r* is very low (*r* = .04, see Figure S2-B), then the null distribution will be shifted to the right. More specifically, there is a higher chance that the predictor loading values, generated from *Z* and the permuted *G* matrix, will be higher than *r =* .04 in both iterations of the split-half (just by chance), and the null distribution will be skewed towards the ceiling value of 1. In this situation, the PLRP values corresponding to barely significant (*p* = .05; blue vertical line) and very significant (*p* = .001; red vertical line) will both correspond to very high PLRP values (see Figure S2B), leading to a collapse of the difference between the cutoffs for barely significant (*p*= .05) and very significant (*p*= .001) *PLRP* values, and this is not optimal for significance testing. Conversely, if the *r*-value is too high (*r* = .45, Figure S2D), then the null distribution is skewed to the left, and again leading to a collapse of the difference between the cutoffs for barely significant (*p* = .05) and very significant (*p* = .001) *PLRP* values, which is also not suitable for significance testing. Heuristically, we define the optimal *r*-value to have a maximum separation between the cutoffs for barely significant (*p* = .05) and very significant (*p* = .001) *PLRP* values (i.e. *PLRP* (*p* = .001) – *PLRP* (*p* = .05)). To find the optimal *PLRP* value cutoffs, we computed *PLRP* (*p* = .001) – *PLRP* (*p* = .05) for a range of *r*-values (from .04 to .45), and determine the optimal *r*-value by plotting this difference on the Y axis and the range of *r*-values along the X axis. As shown in Figure S2A, the optimal *PLRP* value cutoff in the current study was .26 (also see Figure S2C). Although we present the results for *r* = .26, a range of *r*-values around the optimal produced the same set of reliable predictor loadings (*r* in the range of from .22 to .30).

1. **iCPCA Interpretation**

Since both component and predictor loadings represent correlation coefficients (Pearson's *r*), they inherently provide effect sizes. The squared loading value (*r²*) indicates the proportion of variance explained between variables, which is equivalent to the effect size used in analysis of variance ^6^. The mean of all predictor loadings across all the folds/iterations, and the component loadings from the concatenated *GC* matrix, are used as effect sizes to interpret the results. Component loadings reflect the relative importance of each criterion variable within a given component, while predictor loadings indicate the significance of each predictor variable for that component. Because these two types of loadings provide complementary information, they must be interpreted together. Specifically, both are computed as correlations with rotated component scores; however, component loadings are derived from variance-constrained cognitive variables, while predictor loadings are based on symptom rating variables.

**3.1. Identifying the dominant component loadings**

CPCA provides component loadings that indicate the significance of each criterion variable (cognitive measures) within a given component. In traditional PCA, dominant loadings are typically interpreted using an arbitrary threshold. However, with iCPCA, we employ a leave-one-out procedure to assess the contribution of each criterion variable in determining the values of significant predictor variables (identified in the previous step). This process is conducted on the concatenated GC matrix, where the columns represent the criterion variables.

To determine the dominant component loadings, each criterion variable was sequentially regressed out of the remaining criterion variables in the concatenated GC matrix (leave-one-out procedure for cognitive measures), and the component scores were recomputed. PLRP values were then recalculated using the same procedure described earlier (see Figure S3). This step was repeated for each criterion variable, with PLRP values recomputed after removing each one. Criterion variables that, when regressed out, substantially reduced the mean PLRP of significant predictor variables were considered dominant and interpreted accordingly.

To facilitate this interpretation, we ranked all component loadings by dominance (from the greatest to the lowest reduction in PLRP values for each iCPCA component) and examined breaks in dominance, similar to the approach used in scree plots. However, as with PCA, the final selection of component loadings remains somewhat subjective—unlike predictor loadings, which are not subject to this arbitrariness.

**References**

1. Chinchani AM et al. Item-specific overlap between hallucinatory experiences and cognition in the general population: A three-step multivariate analysis of international multi-site data. Cortex. 2021;145(131-144. <https://doi.org/10.1016/j.cortex.2021.08.014>

2. Skiba RM et al. Overlap between individual differences in cognition and symptoms of schizophrenia. Schizophr Res. 2024;270(220-228.

3. Cattell RB. The scree test for the number of factors. Multivariate Behavioral Research. 1966;1(2):245-276. <https://doi.org/10.1207/s15327906mbr0102_10>

4. Cattell RB, Vogelmann S. A comprehensive trial of the scree and kg criteria for determining the number of factors. Multivariate Behavioral Research. 1977;12(3):289-325. <https://doi.org/10.1207/s15327906mbr1203_2>

5. Benjamini Y, Hochberg Y. Controlling the false discovery rate: A practical and powerful approach to multiple testing. Journal of the Royal statistical society: series B (Methodological). 1995;57(1):289-300.

6. Levine TR, Hullett CR. Eta squared, partial eta squared, and misreporting of effect size in communication research. Human Communication Research. 2002;28(4):612-625.

# **Tables**

**Table S1.** The three samples ' MATRICS characteristics, with F and p values from one-way

| **Variable** | **Statistic** | **Canada** | **Australia I** | **Australia II** | **Total** | ***F*** | ***p*-value** |
| --- | --- | --- | --- | --- | --- | --- | --- |
| TMT Part A | Mean | 38.93 | 31.74 | 46.47 | 37.79 | 2.70 | .07 |
|  | SD | 15.30 | 12.50 | 67.24 | 37.26 |  |  |
| DCS | Mean | 40.29 | 45.95 | 40.37 | 42.83 | 6.11 | **<.01** |
|  | SD | 12.22 | 10.37 | 11.59 | 11.54 |  |  |
| CPT-IP 2-digt | Mean | 2.71 | 3.12 | 2.61 | 2.86 | 4.81 | **.01** |
|  | SD | 1.06 | 0.91 | 1.23 | 1.07 |  |  |
| CPT-IP 3-digt | Mean | 1.99 | 2.33 | 1.82 | 2.10 | 4.59 | **.01** |
|  | SD | 1.15 | 0.97 | 1.00 | 1.05 |  |  |
| CPT-IP 4-digt | Mean | 1.05 | 1.28 | 0.93 | 1.12 | 3.13 | **.05** |
|  | SD | 0.73 | 0.94 | 0.82 | 0.86 |  |  |
| SS forward | Mean | 7.91 | 7.53 | 7.48 | 7.63 | 1.01 | .37 |
|  | SD | 1.80 | 1.77 | 1.79 | 1.78 |  |  |
| SS backwards | Mean | 6.71 | 7.30 | 6.43 | 6.89 | 3.26 | **.04** |
|  | SD | 2.02 | 2.08 | 2.09 | 2.09 |  |  |
| LNS | Mean | 11.27 | 13.44 | 11.46 | 12.29 | 7.92 | **<.001** |
|  | SD | 3.93 | 3.14 | 4.14 | 3.79 |  |  |
| HVLT trial 1 | Mean | 5.05 | 5.63 | 3.80 | 4.96 | 15.76 | **<.001** |
|  | SD | 1.94 | 1.80 | 1.98 | 2.03 |  |  |
| HVLT trial 2 | Mean | 6.98 | 7.44 | 5.59 | 6.81 | 12.45 | **<.001** |
|  | SD | 2.13 | 2.14 | 2.24 | 2.29 |  |  |
| HVLT trial 3 | Mean | 8.16 | 8.44 | 6.44 | 7.82 | 10.98 | **<.001** |
|  | SD | 2.28 | 2.41 | 3.00 | 2.68 |  |  |
| BVMTR trial 1 | Mean | 3.44 | 3.51 | 3.44 | 3.32 | 1.26 | .29 |
|  | SD | 2.48 | 2.50 | 2.48 | 2.38 |  |  |
| BVMTR trial 2 | Mean | 5.07 | 5.83 | 5.46 | 5.52 | 1.02 | .36 |
|  | SD | 2.90 | 3.16 | 3.27 | 3.12 |  |  |
| BVMTR trial 3 | Mean | 6.41 | 7.28 | 6.24 | 6.75 | 1.86 | .16 |
|  | SD | 3.27 | 3.47 | 3.78 | 3.52 |  |  |
| Mazes | Mean | 13.55 | 13.63 | 13.22 | 13.49 | 0.06 | .94 |
|  | SD | 6.88 | 6.85 | 7.00 | 6.87 |  |  |

*Note: Significant p-values are bold. Abbreviations: Brief Visuospatial Memory Test-Revised (BVMTR), Continuous Performance Task Identical Pairs (CPT-IP), Digit Symbol Coding (DSC), Hopkins Verbal Learning Task (HVLT), Letter-Number Sequence (LNS), Spatial Span (SS), Trail Making Test (TMT).*

**Table S2**. Scheffé post-Hoc analysis for significant results from Table S1

| **Variable** | **Pair Comparison** | **Mean Difference** | **Standard Error** | **Scheffé p-value** |
| --- | --- | --- | --- | --- |
| **DCS** | Canada vs Australia I | 5.66 | 2.00 | <.01 |
|  | Australia I vs II | 5.58 | 1.93 | <.01 |
| **CPT-IP 2-digit** | Canada vs Australia I | 0.41 | 0.17 | .02 |
|  | Australia I vs II | 0.51 | 0.19 | <.01 |
| **CPT-IP 3-digit** | Australia I vs II | 0.51 | 0.17 | <.01 |
| **CPT-IP 4-digit** | Australia I vs II | 0.35 | 0.15 | .02 |
| **SS backwards** | Australia I vs II | 0.87 | 0.36 | .02 |
| **LNS** | Canada vs Australia I | 2.17 | 0.63 | <.01 |
|  | Australia I vs II | 1.98 | 0.66 | <.01 |
| **HVLT trial 1** | Canada vs Australia II | 1.25 | 0.38 | <.01 |
|  | Australia I vs Australia II | 1.83 | 0.33 | <.001 |
| **HVLT trial 2** | Canada vs Australia II | 1.39 | 0.42 | <.01 |
|  | Australia I vs Australia II | 1.85 | 0.38 | <.001 |
| **HVLT trial 3** | Canada vs Australia II | 1.72 | 0.51 | <.01 |
|  | Australia I vs Australia II | 2.00 | 0.48 | <.001 |

*Note: Only significant pairs are presented in the table.*

# **Figures**

**Figure S1.** Diagram explaining steps involved in iCPCA. A) In each split-half iteration, the cognitive measures (criterion variables, *Z*) and symptom items (predictor variables, *G*) were randomly split into two halves: (*Z_1_, G_1_*) and (*Z_2_, G_2_*). CPCA was performed on both halves independently to obtain *GC* matrices. This was repeated for 1000 iterations. B) *GC* matrices for both the halves and all 1000 iterations were vertically concatenated. PCA was performed on the concatenated *GC* matrix to obtain common component loadings (*L*) and concatenated component scores (*S*) that were split and correlated with the corresponding *G* matrices to obtain predictor loadings (*P*) for each fold/iteration. Predictor loadings were averaged across folds/iterations to obtain mean predictor loadings (mean *P*). Predictor loading reliability proportions (*PLRP*s) were computed, for each element (*r*) in the *P* matrix, as the proportion of iterations for which the predictor loadings (*P*) in both the split halves was greater than or equal to 0.26 (optimized *r* value for this study, see section **2. Significance test and optimization of the hyperparameter (*r*)** and Figure S2.


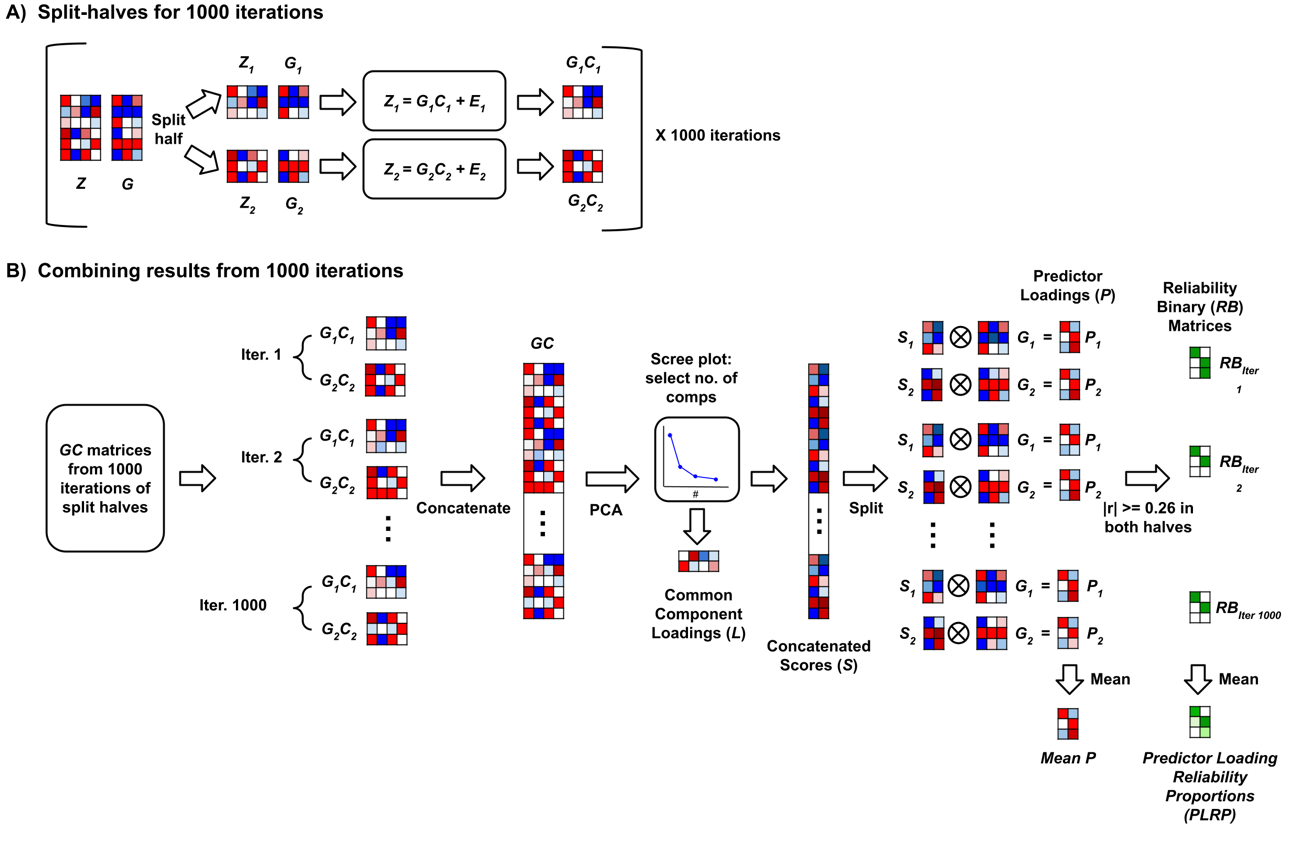


**Figure S2.** Optimization of the hyperparameter (*r*) based on predictor loading reliability proportions (PLRP), computed as the proportion of iterations that showed significant predictor loadings in both split-half solutions. A) Plot of *PLRP* (*p*=0.001) – *PLRP* (*p*=0.05) vs *r*-values. We defined optimal *r*-value to have a maximum separation between barely significant (*p* = .05) and significant (*p*= .001) *PLRP* values. For the current dataset, we obtained an optimal *r*=0.26 (Black vertical line). B-D) Null distributions were obtained using very liberal (B), optimal (C), and very conservative (D) *r*-values. Blue and red vertical lines represent the barely significant (*p* = .05) and very significant (*p*=0.001) *PLRP* values, respectively.


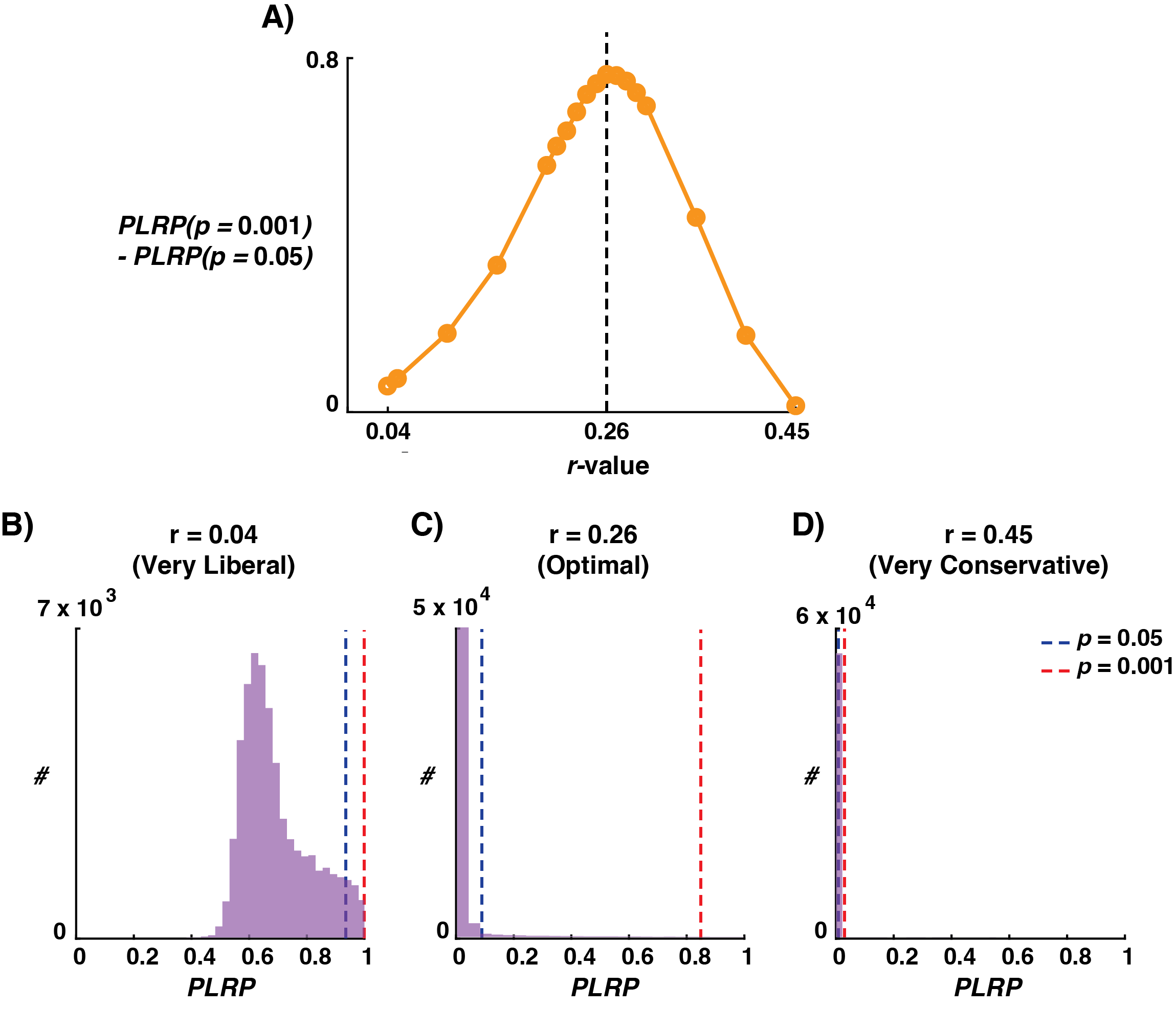


**Figure S3** Scree plot used in the process of component selection. We extracted two components.

**
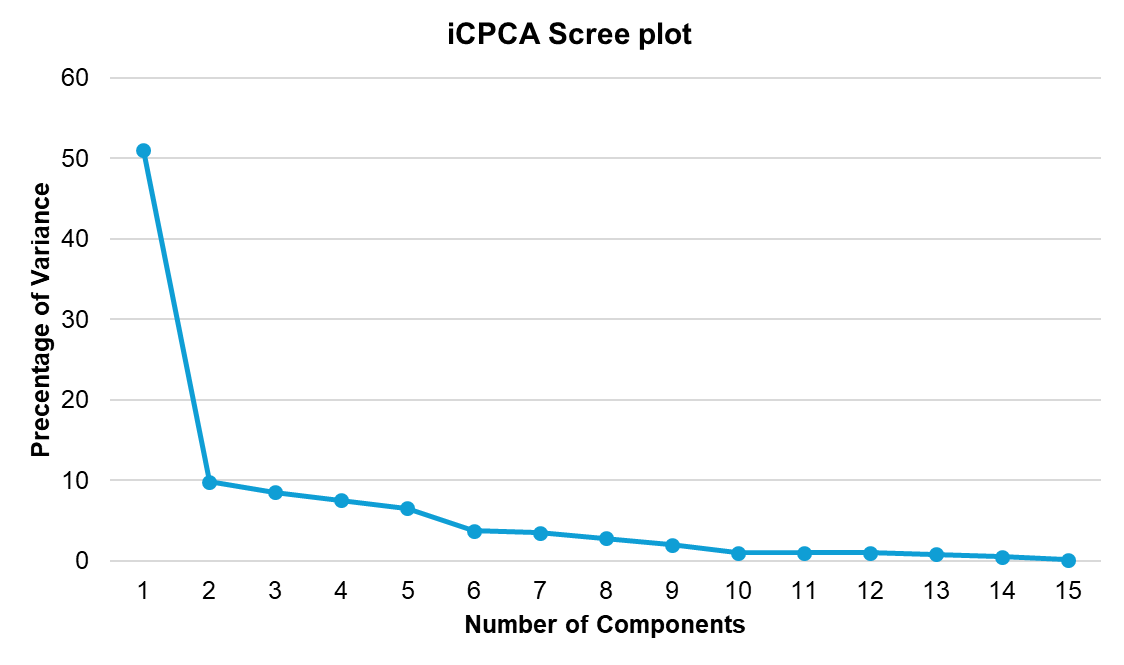
**

**Figure S4.** Average predictor loading reliability proportions (*PLRPs).* Average predictor loading reliability proportions (*PLRPs)* were obtained by regressing each criterion variable out of the remaining criterion variables for Components 1 (subpanel A) and 2 (B) separately. For example, in the case of Component 1, regressing *HVTL immediate* out of all other criterion variables resulted in a reduction in *PLRP* value averaged over all predictor loadings (the ones reliable in the primary analysis) to essentially zero, suggesting that this variable is essential to the dimensional structure of the results. Using a criterion like component selection in a scree plot, we retained the first three variables as dominant component loadings for Component 1. In the case of Component 2, we retained the first four variables. *Abbreviations: Brief Visuospatial Memory Test-Revised BVMTR), Continuous Performance Task Identical Pairs (CPT-IP), Digit Symbol Coding (DSC), Hopkins Verbal Learning Task (HVLT), Letter-Number Sequence (LNS), Spatial Span (SS), Trail Making Test (TMT).*


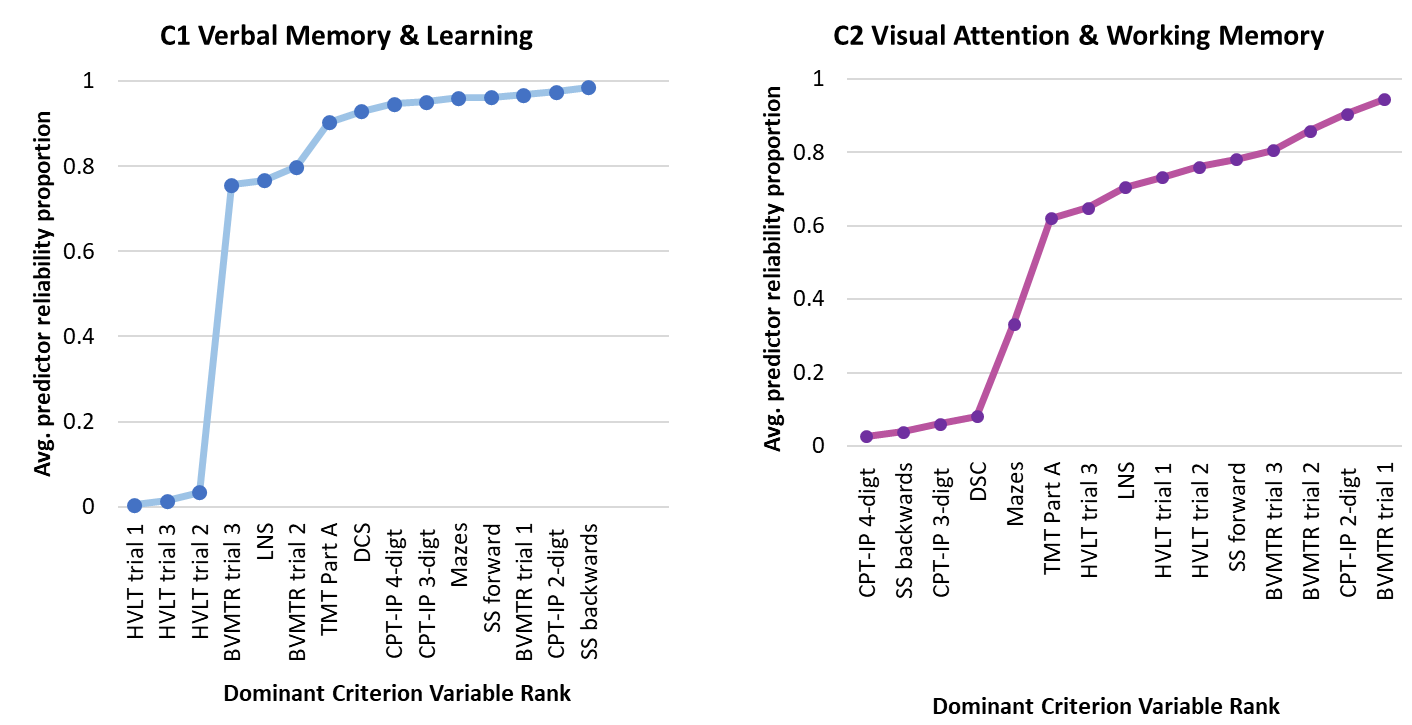

Supplement: sj-docx-1-cpa-10.1177_07067437251387565 - Supplemental material for A multisite study of the overlap between symptoms and cognition in schizophrenia: Une étude multicentrique sur le chevauchement entre les symptômes et les troubles cognitifs chez les personnes atteintes de schizophrénie [file sj-docx-1-cpa-10.1177_07067437251387565.docx]
